# Supplementary figures and images for: A model of Notch signalling control of angiogenesis: Evidence of a role for Notch ligand heterodimerization
Source: PLoS Comput Biol. 2025 Feb 11;21(2):e1012825. doi: 10.1371/journal.pcbi.1012825 (PMC11841921; doi:10.1371/journal.pcbi.1012825)

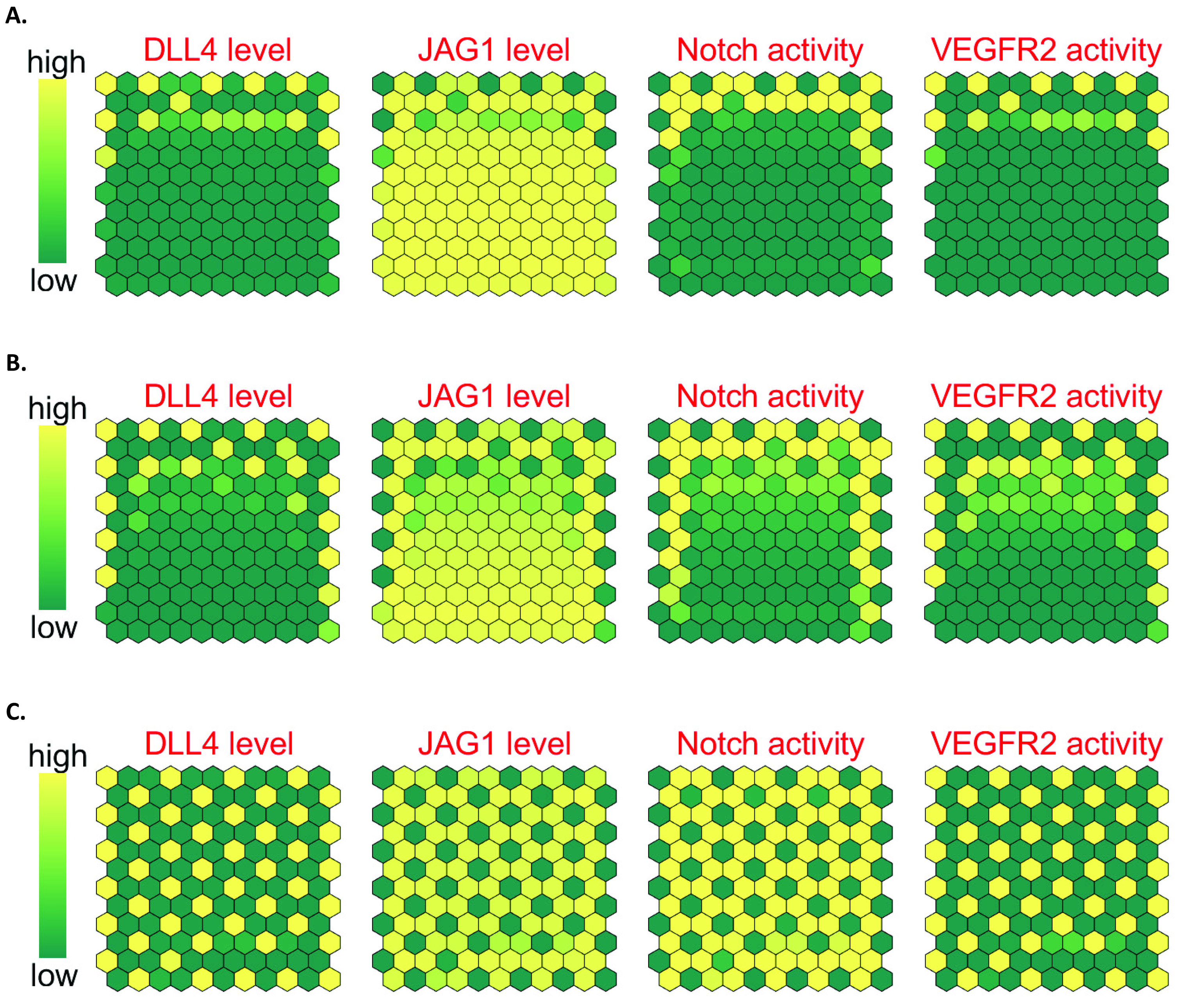

Supplement: S6 Fig — The simulations were performed with a shallower VEGF gradient. The VEGF concentration was set according to: (A) Vy=Vext/exp2y−y0; (B) Vy=Vext/exp5y−y0; C) Vy=Vext/exp35y−y0. See Numerical simulations (Methods) for simulation information. (TIF) [file pcbi.1012825.s006.tif]

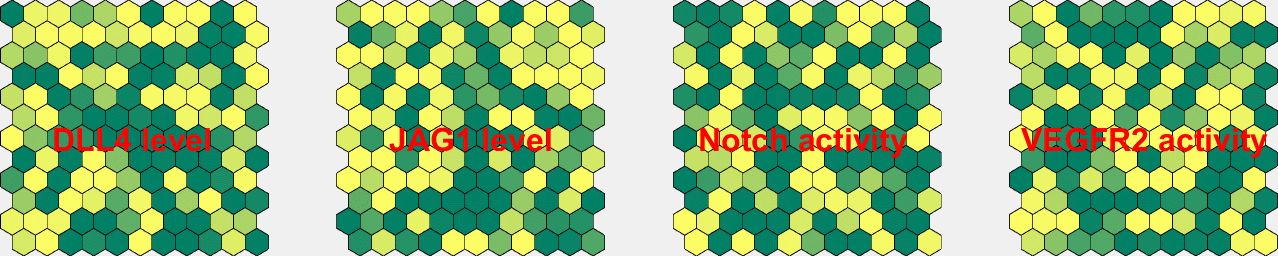

Supplement: S1 Video — This video corresponds to Fig 5A with DLL4-JAG1 heterodimerization. (GIF) [file pcbi.1012825.s011.gif]

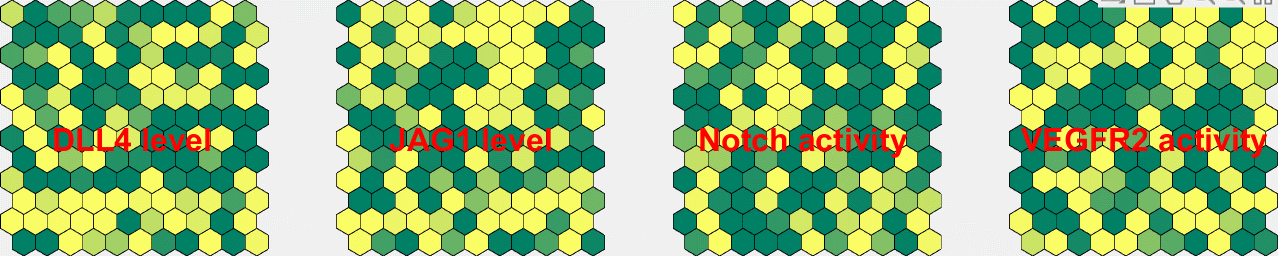

Supplement: S2 Video — This video corresponds to Fig 5B without DLL4-JAG1 heterodimerization. (GIF) [file pcbi.1012825.s012.gif]
