# Supplementary material for: A model of Notch signalling control of angiogenesis: Evidence of a role for Notch ligand heterodimerization
Source: PLoS Comput Biol. 2025 Feb 11;21(2):e1012825. doi: 10.1371/journal.pcbi.1012825 (PMC11841921; doi:10.1371/journal.pcbi.1012825)
Supplement: Attachment — Submitted filename: Chen et al. REVISION Cover letter 10.12.24.docx [file pcbi.1012825.s014.docx]

**David Baker PhD**
Department of Cell & Chemical Biology,

Leiden University Medical Center (LUMC)
2300 RC Leiden
The Netherlands
email: [d.baker@lumc.nl](mailto:d.baker@lumc.nl)

Tel: +31 71 526 9223

+31(0) 641726068

Wednesday 11^th^ December, 2024.

Dear Professor Faeder & Professor Haugh,

We would be most grateful if you would consider the accompanying revised manuscript for publication as an Article in PLOS Computational Biology. We were delighted with your positive assessment of our paper and that the three reviewers were very supportive of our work - we thank them for their helpful remarks and their excellent suggestions for amending our manuscript. We have comprehensively addressed these issues yielding, we believe, a substantively improved manuscript.

In light of the reviewers’ comments, we remain confident that our work will be of broad appeal to both the scientific and clinical community across multiple disciplines including cell biology, physiology and systems biology, and pathologies thereof.

We very much hope that our manuscript is now suitable for publication. Should you require any further information, please do not hesitate to contact me.

Our response to each of the reviewers’ remarks are detailed below. Two versions of the manuscript are provided: an unmarked version and one in which changes have been highlighted in **blue**.

We look forward to hearing from you.

Yours sincerely,

David Baker

*Dept. Cell & Chemical Biology,*

*Leiden University Medical Center (LUMC)
Leiden
The Netherlands.
Email:* [d.baker@lumc.nl](mailto:d.baker@lumc.nl)

Tel: +31 (0) 641726068

**REVIEWER 1**

We thank the reviewer for the favourable assessment of our manuscript and the careful summary of the importance/significance of our work. They raised a number of relevant and interesting questions we have addressed below.

**1. The authors study experimentally the capacity of DLL4, JAG1 and JAG2 to trans-activate and cis-inhibit Notch1. They show that JAG1 does not promote trans activation or cis inhibition. They then explain the first observation by lack of binding and the second observation by lack of dimer formation. Can the lack of binding itself be enough to explain both observations? While they compare the cis behavior to that of JAG2, there is no comparison of the trans behavior.**

**Response:** Regarding, the first part of the question, the reviewer is correct that our evidence supports the view that JAG1 inefficiently trans-activates Notch1 (Fig 1A), and a plausible explanation for this is the relatively low affinity of trans JAG1/Notch interactions (Fig 1B). We further show that JAG1, by comparison with DLL4, is relatively ineffective at promoting cis-inhibition (Fig 2A), and, as suggested by the reviewer, one possible explanation for this could be a lack of binding to the receptor in-cis. We favour the view that the relative lack of JAG1 mediated cis-inhibition results from its inability to forms dimers. We have recently shown that ligand dimerization is necessary for cis-inhibition (ref 15), and here we show, in contrast to all other Notch ligands, that JAG1 cannot multimerize (Fig 2D). We also identify the motif in JAG2 which enables JAG2 dimerization (and whose absence in JAG1 precludes JAG1 dimerization), and deletion of this motif blocks JAG2 dimerization (Fig 2D) and correspondingly blocks efficient JAG2-mediated cis-inhibition to the same degree as JAG1 (Fig 2E). Moreover, when JAG1 (and indeed other ligands) and Notch are expressed in the same cell (in cis), they can relatively efficiently associate (the precise conformational differences between trans Notch-ligand binding and cis Notch-ligand are currently unknown- it would be of considerable interest to the field to delineate the differences). We have added this additional data as supplementary Fig S1 and modified the text to clarify this viewpoint.

Regarding, the second part of the question, we did compare JAG1 and JAG2 trans activation but did not include it as our focus was cis inhibition. We agree with the reviewer that the data can be included without disrupting the flow of our argument and have now adjusted Fig 1A accordingly.

**2. In the model, the author uses a single degradation parameter for all ligand monomers, dimers, and the Notch receptor. What is the motivation behind this assumption? How valid is it? Can this affect the results?**

**Response:** This is a good point. In the model, the assumed degradation rates represent an average level of most proteins (see Methods). To further test the validity of this assumption, we have added a new Figure S5 in which we simulated the effects of varying degradation rates on Notch activity by using different degradation rates for ligand monomers, ligand dimers and Notch receptors. The simulations show that our results do not change with different degradation rates.

**3. The model nicely describes the binding of ligands and receptors. However, it completely ignores possible reverse reactions through which complexes revert to their monomeric components. Critically, this allows the authors to solve the first two equations in (1) for D and J independently of the number of complexes or receptors. What is the justification for this assumption, and how would the result change if unbinding were allowed to occur?**

**Response:** Thank you for this valid point. We have clarified the basis for this assumption in lines 209-210 and have discussed it in lines 456-461. Indeed, the dissociation of the DLL4 homodimer makes it impossible to solve the first two equations in model (1) independently of other variables. To further illustrate the justification for this assumption, we have added more mathematical analysis in the Methods (see lines 531-550) and a new Figure S3 B in the Supplementary information to show that the results would not change if the disassociation of cis-complexes could occur in our model.

**Minor points:**

**4. While all the information is in the manuscript, writing more information within the figures could ease the reading.**

**Response:** We agree with the reviewer and have added some additional information to enable a clearer understanding of the data.

**- The immunoprecipitation panels could be more readable by explicitly stating the antibodies used for precipitation and staining.**

**Response:** We agree with the reviewer and have fully annotated our presented experimental data to enable a clearer understanding of the data.

**- The legends show the Notch receptor as a single protein, but on the cells, two receptors bind to a single ligand. This is confusing, as the model considers a 1-1 binding between a single receptor and a single ligand.**

**Response:** We have modified the schematic diagrams/models to make this point clearer.

**- The cartoons for the sprouting angiogenesis simulations could gain from another panel of the in-vivo expected result for comparison.**

**Response:** We have included a new Fig S7 A which schematically depicts the opposing effects of DLL4 and JAG1 on *in vivo* sprouting angiogenesis.

**- Line 251: there might be a missing square root. The condition for B2 to be a small number is k_he >> \sqrt(k_ho).**

**Response:** We thank the reviewer for highlighting this error, which we have corrected (see line 256).

**REVIEWER 2**

We thank the reviewer for their positive assessment of our work. They raised a number of relevant and interesting questions we have addressed below.

**1. I feel that the use of "A new model of Notch signalling", as in the previous paper, is a bit of an overstatement.**

**Response:** We agree with the reviewer and have provided an alternative title: A model of Notch signalling control of angiogenesis: evidence of a role for ligand heterodimerization.

**2. I also feel that calling one's own previous recent paper "seminal" is a bit much...**

**Response:** We thank the reviewer for such a polite rebuke. This was indeed a particularly embarrassing faux pas on our part- the citation for this statement should have been Benedito et al (18) not our own earlier work (cited in error). Humble apologies!

**3. The authors should provide a bit more information about the simulations. For Fig 5A, B, where exactly is y = y_0 (used in the exponential decay function)? It is not clear. Also, how exactly does the coordinate y relate to the cell rows? It is not clear in the manuscript. For the simulations, what were the initial and boundary conditions used? As it is, the model shows a lateral inhibition-like pattern in row 1 (sometimes also row 2);** **would this emerge in more rows of cells for a shallower VEGF gradient? It is also not clear how that pattern of Notch signalling activity translates to the biology of sprouting angiogenesis. I understand that this corresponds to the selection of tip cells, but is** **the period-2 spacing appropriate for the real system? Is the initial pattern of Notch activity period-2 in reality, or is it longer-range (as is typically the case?) A bit** **more information to link the simulations to the target system would be helpful.**

**Response:** We thank the reviewer for raising these points. We have provided more information about the simulations, including the gradient of VEGF concentrations and initial boundary conditions (see lines 582-588). In Fig 5A-B, we assumed a sharp gradient of VEGF concentration to mimic the experimental observations where VEGF-induced tip cells are always formed at the leading edge of a sprouting vessel. In a new Figure S6, our simulations show that the mentioned lateral-inhibition-like pattern in row 1 would emerge in more rows of cells for a shallower VEGF gradient. The reviewer correctly points out that in our model the relationship between cell signaling and sprouting angiogenesis is linked through tip cell formation, and in this context, the tip cell pattern with period-2 spacing is appropriate for the *in vivo* observations in sprouting angiogenesis where, during the phase of tip cell selection, the leading edge will be composed of tip cells and neighbouring stalk cells. As requested, we have provided more information (see lines 324-325, lines 356-358) to link more clearly the simulations to sprouting angiogenesis.

**4. Fig. 4 shows slices through the (b_D, b_J) plane. Would it be possible to present a full picture of NICD across the plane (a 2D plot)? I think I know what it will look like, but it would be good to see how NICD depends on the full range of production rates.**

**Response:** We thank the reviewer for this valid point. We agree and have added a new figure S4 to show the dependence of NICD on the full range of DLL4/JAG1 production rates.

**5. In this model, as with the Sprinzak model of cis- and trans- binding, binding is assumed to be irreversible. So Notch and its ligands form cis- complexes that never dissociate. I think it would be good to state this assumption. I presume those complexes are going somewhere. Should I assume they are degraded / endocytosed?**

**Response:** We thank the reviewer for highlighting this point (which was also raised by Reviewer 1). Indeed, the cis-complexes are assumed to be inactive and were assumed to be degraded in the cell. We have stated the assumption in lines 209-210 and have provided more discussion (see lines 456-461, lines 531-550) and additional simulations to clarify this issue (see Fig S3 B), which shows that the results would not change if the disassociation of cis-complexes could occur in our model.

**6. Equation (6) is really an approximation (the authors use the expression "reduces to", but that would strictly only be in the limit). I think it should be written as such.**

**Response:** We agree and have adjusted the text, as recommended.

**7. On line 343, I think the statement that the model "correctly predicts" is an overstatement; that relationship between VEGFR2 activity and high DLL4 expression is put in the model (the production rate of DLL4 is an increasing Hill function of VEGFR2 activity...).**

**Response:** We agree with the reviewer and have adjusted the text accordingly.

**8. The use of "expression" on line 346 is unhelpfully vague. I can't work out what the authors mean here. I think expression refers to the steady state amount of free ligands in the plasma membrane, since the production rates of ligands (what one might expect to be meant by "expression") are spatially uniform. This may seem a bit semantic, but I think it is important to be clear here, since it is at the heart of the proposed patterning mechanism.**

**Response:** Many thanks for pointing this out. We agree with the reviewer and have adjusted the text accordingly.

**9. A couple of minor typos: On line 455, "principle" should be "principal". In Table 1, "typic" should be "typical"**

**Response:** We thank the reviewer for highlighting these errors, which we have corrected.

**REVIEWER 3**

We thank the reviewer for their favourable and precise summary of our work. They raised a number of relevant and interesting points we have addressed below.

**1. At which cell confluency was the cis-inhibition experiments performed? The authors should at least briefly explain whether they would expect co-expressing ligands in the “receiver” cells to increase the transactivation between two adjacent “receiver” cells and whether they considered the cell confluency as a factor that can convolute the experimental results.**

**Response:** Cis inhibition assays were performed using monolayers of mixed sender-receiver cultures at approximately 90% confluency (0.45 x 10^6^ cells/well), to ensure cells were touching to enable suitable transactivation. We initially optimized these assays by testing different ratios of receiver:sender cells (1:1, 1:2) and found no obvious influence on outcome, thus we routinely seeded receiver/sender cells in 1:1 ratios (we also perform the experiments using ‘receiver’ cells stimulated by recombinant ligands, rather than co-cultured cells, and obtain comparable results). We have further clarified these points in the Methods.

**2. In general, figures with Western blot images need further clarification and addition of the necessary controls. Please see below:
In Figure 1D, the size of all three Notch paralogs seems to be the same, even though the Notch3 receptor should be about 20kD smaller. The authors should explain what this band corresponds to and perhaps show the full membrane together with the protein ladder in the supplementary data. The size of the bands, and blots of an appropriate housekeeping protein should be added. Additionally, the blots are mis-aligned and do not match the sample labels properly.**

**Response:** We agree with the reviewer and have annotated our presented experimental data to enable a clearer understanding of the data. Regarding Fig 1D, we have further clarified what the bands correspond to and include fuller blots and molecular weight markers. It is true that the mass of N3 is predicted to be approximately 20 kDa smaller than N1 and N2, however, these complexes are very large >300 kDa, not least due to the chemical crosslinking step used in this assay, and such a mass difference could not be resolved even using 6% polyacrylamide gels (in the absence of crosslinking, if the gel is run for an extended period, it can be possible to discern the mass difference e.g. Fig S1). The blots are uniformly ‘clean’ since these assays are performed at 4^0^C to stabilize receptor and ligand at the cell membrane, which are subsequently immunopurified and detected by Western blotting, thus under these conditions we would not expect to (easily) detect processed forms of Notch. Regarding the use of a housekeeping gene, in this assay the crucial control is the total amount of receptor expressed, since relative ligand binding is related to the total amount of receptor present, which we first optimized to ensure (approximately) equal levels of expression for each condition- we have clarified this point in the legend. The reviewer is correct with regard to label misalignment, which we have adjusted.

**3. While how the receptor and the ligand constructs were tagged were explained in the methods section, it is difficult to understand the construct identity this by looking at the figure, since only the tag names are shown. Adding this information to the figures either in the cartoons or on the blots is recommended.**

**Response:** The reviewer is correct and we have adjusted all blots accordingly.

**4. Figure 2: The blots from the immunoprecipitation experiments are difficult to understand. The authors should annotate the blots with the information which proteins were pulled down and what they were probed for afterwards (e.g. IP:His, WB: His and IP:His, WB:HA) to make them easier for the reader to understand.**

**Response:** The reviewer is correct and we have adjusted all blots accordingly.

**5. Figure 5S: Considering the Notch1 construct was tagged in the C-terminal, wouldn´t the authors expect to see the cleavage products of the Notch receptor in their blots when it is activated by the ligand-expressing cells? Judging by the Dll4, Jagged1, Notch1 co-blots the authors are only showing the full length Notch1 precursor. Additionally, the authors should again annotate the protein size on these blots, and possibly blot the same samples for the cleaved NICD to further support the reporter assay results if it is not possible to visualize the cleavage products otherwise.**

**Response:** As requested we have added molecular weight markers to the blots. We included (and routinely always include) Western blots of the actual luciferase reporter lysates essentially to ensure that proteins are actually expressed and to show the relative protein levels under each condition (frustratingly, something we often fail to see in the published literature). However, under these conditions (small scale cultures, luciferase cell lysis conditions/lysis buffer), and particularly for Notch1 whose ICD is the most difficult to detect compared to other Notch paralogues, we do not routinely detect processed Notch fragments, either using antibodies directed against the epitope tag or the Notch1 intra antibody (Cell Signaling). However, using appropriate conditions we can routinely demonstrate Notch cleavage. For the crucial experiment shown in Fig 3E, which shows that JAG1 relieves DLL4-mediated cis-inhibition by direct binding to cis-DLL4, thus enabling DLL4-dependent trans-activation of Notch, we performed the experiment to monitor Notch receptor cleavage. For this, cells were plated at higher cell density (6 cm dishes) and lysed directly in denaturing Laemmli buffer. We show efficient DLL4-dependent Notch cleavage is blocked by co-expression of cis-DLL4 (due to cis-inhibition), which can be relieved by co-expression of JAG1 and DLL4, in cis. Consistent with the reporter assay, JAG1 cannot relieve DLL4-mediated cis inhibition of a mutant DLL4 protein that is unable to form JAG1-DLL4 heterodimers. We have included an additional supplementary Fig (Fig S2) of this data.

**6. Line 832, 836, 851, 854: “tissue-culture cells” sounds off. Please revise.**

**Response:** We have revised the text accordingly.

**7. There seems to be major issues relating to the coherence of the manuscript. For example, the use of the Notch3 ECD protein and of DAPT as a control condition for the luciferase assays were elusive in the text and in the figures, even though they were mentioned in the material and methods section(?). The authors must make sure that the writing is consistent with the figures.**

**Response:** The reviewer is correct and we have adjusted all the text accordingly. Regarding the specific example used, we must apologize for this careless oversight: another Notch manuscript was being prepared at the same, which does include certain common methodologies and unfortunately appropriate editing was missing in this instance- we have corrected this mistake by removing extraneous references.

**8. For all figures, the appropriate statistic tests should be done for the experimental data, and corresponding graphs should be annotated with the results.**

**Response:** We have performed the relevant statistical tests and revised the graphs accordingly.
